# Transcriptomics and Metabonomics Identify Essential Metabolic Signatures in Calorie Restriction (CR) Regulation across Multiple Mouse Strains

**DOI:** 10.3390/metabo3040881

**Published:** 2013-10-11

**Authors:** Sebastiano Collino, François-Pierre J. Martin, Ivan Montoliu, Jamie L. Barger, Laeticia Da Silva, Tomas A. Prolla, Richard Weindruch, Sunil Kochhar

**Affiliations:** 1 Nestlé Institute of Health Sciences SA, Molecular Biomarkers Core, Campus EPFL, Quartier de l’innovation, bâtiment H, Lausanne 1015, Switzerland; E-Mail: Laeticia.DaSilva@rd.nestle.com; 2 Nestlé Research Center, Nestec Ltd., Vers-chez-les-Blanc, CH-1000 Lausanne 26, Switzerland; E-Mails: Ivan.MontoliuRoura@rdls.nestle.com (I.M.); sunil.kochhar@rd.nestle.com (S.K.); 3 LifeGen Technologies, LLC (Limited Liability Company), Madison, WI 53719, USA; E-Mail: jamie.l.barger@gmail.com; 4 Departments of Genetics and Medical Genetics, University of Wisconsin, Madison, WI 53704, USA; E-Mail: taprolla@wisc.edu; 5 Department of Medicine, University of Wisconsin and William S. Middleton Veteran’s Hospital, Madison, WI 53705, USA; E-Mail: rhweindr@wisc.edu

**Keywords:** metabolomics, healthy ageing, calorie restriction, lipidomics

## Abstract

Calorie restriction (CR) has long been used to study lifespan effects and oppose the development of a broad array of age-related biological and pathological changes (increase healthspan). Yet, a comprehensive comparison of the metabolic phenotype across different genetic backgrounds to identify common metabolic markers affected by CR is still lacking. Using a system biology approach comprising metabonomics and liver transcriptomics we revealed the effect of CR across multiple mouse strains (129S1/SvlmJ, C57BL6/J, C3H/HeJ, CBA/J, DBA/2J, JC3F1/J). Oligonucleotide microarrays identified 76 genes as differentially expressed in all six strains confirmed. These genes were subjected to quantitative RT-PCR analysis in the C57BL/6J mouse strain, and a CR-induced change expression was confirmed for 14 genes. To fully depict the metabolic pathways affected by CR and complement the changes observed through differential gene expression, the metabolome of C57BL6/J was further characterized in liver tissues, urine and plasma levels using a combination or targeted mass spectrometry and proton nuclear magnetic resonance spectroscopy. Overall, our integrated approach commonly confirms that energy metabolism, stress response, lipids regulators and the insulin/IGF-1 are key determinants factors involved in CR regulation.

## 1. Introduction

Managing the quality of life and delaying the onsets of aging related chronic disease is one of the quests to promote healthy aging. Within this aim, a significant proportion of research into this area is engaged in revealing the mechanisms underlying the aging and/or longevity processes and how such could be modulated. Aging is characterized by an increasing chronic, low-grade inflammatory status indicated as inflamm-aging [1,2] responsible for the major inflammation-driven age-related diseases, such as cardiovascular disease (CVD), diabetes mellitus (DM), Alzheimer disease (AD), and cancer [3,4]. Dietary caloric restriction (CR) is a robust but severe nutritional intervention with plausible effects on healthspan, body function and longevity [5]. Unlike many interventions, life-long CR suggests alteration of fundamental biological processes that control aging [6], as observed in a diverse range of organisms [7,8], including nematodes, mice, rats, dogs [9,10] and/or humans [11]. Both the duration and the time of CR initiation have been found to be crucial for developing an anti-aging strategy. Long-term CR initiated before mid-life has been reported to slow down the aging process and to increase life span in rodents [12] and dogs [13] whereas CR initiated late in life increases mortality in several rodent models [14]. Humans are not likely to benefit from CR as much as these organism models for increased lifespan since they have evolved to minimize the effect of food shortage [15]. However, CR may be a useful anti-aging (increase healthspan) strategy for humans to decrease or delay the onset of degenerative diseases as suggested by studies on non-human primates and human beings [16,17]. Studies of Okinawan centenarians support the view that a low-calorie diet can increase prospects for good health and longevity in humans [17,18]. Findings from the small group of volunteers confined to Biosphere 2 confirmed that a 30% dietary restriction could be imposed for two years and would produce many of the physiological, hormonal, and morphological effects expected [19]. Therefore, identification of pathways and biomarkers activated by caloric restriction would highly contribute to the determination of nutritional strategies aiming to mimic the health benefits of CR. Indeed, CR mimetics provide a more realistic anti-aging strategy by modulating the energy metabolism towards the one observed in CR without the requirement for reduced food intake and to promote healthspan effects. CR mimetics at present include glycolytic inhibitors, stress response enhancers, sirtuin controllers, manipulation of the insulin/IGF-1, lipids and adipokines regulators, and autophagic enhancers [20].

Nowadays the diet-restricted rodent model is widely used to understand the mechanisms of the aging process [21].Yet the underlying the molecular mechanism of the extended life span by CR is still a matter of debate [22,23]. Moreover, despite CR resulting in similar benefits in various animal models, the impact on the physiology and metabolic adaptation to the new metabolic homeostasis varies greatly from one animal model/strain to another [24]. It is therefore vital to delineate key regulatory processes and common metabolic pathways across species translating such knowledge into drug and/or nutritional targets for CR mimetics. Within this quest, metabolic phenotyping is a useful tool to establish gradual metabolic changes linked to dietary intervention and disease development. Recently, metabonomics had successfully been applied to study the modulation of the aging processes following nutritional interventions, including caloric restriction-induced metabolic changes in mouse [25] dogs [26,27], and non-human primates [28] as well as high fat induced weight gain and metabolic disorders [29]. Yet, beyond the insight provided by these studies, a comprehensive and comparative capture of common metabolic functions across multiple animal species is missing. In the present study, liver tissue transcriptomics and blood plasma metabonomics were employed to capture the hepatic and systemic metabolic processes under the influence of CR initiated early in life across six mouse models. We herein report tissue-specific candidate biomarkers that were commonly affected by CR. To fully depict the metabolic pathways affected by CR and confirm the transcriptomics changes, the metabotypes of C57BL6/J mice were further characterized using nuclear magnetic resonance (^1^H-NMR) profiling approach in liver and urine. Among the selected strains of animals, the C57BL6/J mouse model is particularly interesting model due to its higher susceptibility to weight gain under specific dietary conditions due to its genetic background [30]. Indeed C57BL6/J mice fed with a high fat diet develop obesity, mild to moderate glycemia and hyper-insulinemia and related disorders [31]. Moreover, this strain of mice is a well-accepted system model to investigate the nutrition-metabolism paradigm and, therefore, well suited to investigate CR diet.

## 2. Results

### 2.1. CR Led in a Significant Modulation of Body Weight Gain across the Animal Strains

**Figure 1:**
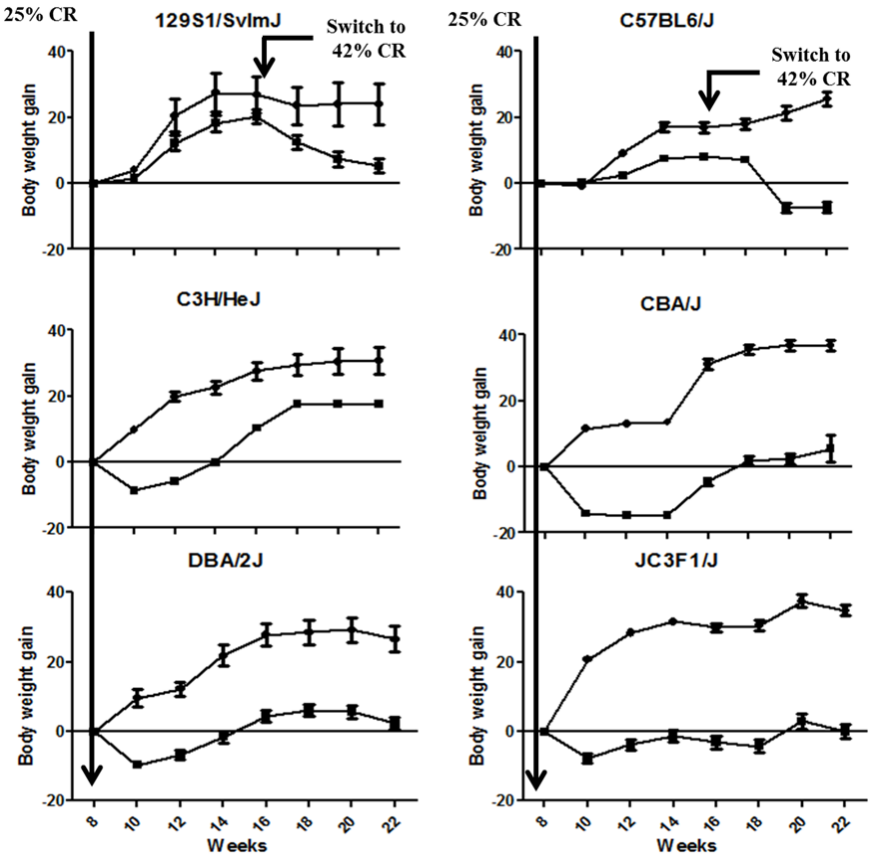
Body weight gain overtime for the different strains of mice under control diet or calorie restriction (CR). Data are reported as mean ± SE values for the body weight gain calculated from Week 8 onwards. Control and CR groups are plotted with dot and square shapes, respectively.

Body weight gain was significantly lowered in all the animal strains by CR (Figure 1, Table A1). Differences of body weight gain were statistically significant between CR and the control group across the different mouse strains, except for 129S1/SvlmJ (only at Weeks 20 and 22) and C57BL6/J strains (from Week 12 to Week 22 only) as tested by ANOVA and Bonferroni post-tests. In particular, body weight was significantly lower in C57BL6/J starting at 20 weeks, which suggests a late response to CR that may be related to the specific genetic susceptibility of this animal strain to gain weight (31). This observation highlights the existence of a strong phenotypic variability within the different mice models and suggests the existence of specific metabolic signatures associated to these CR phenotypes.

### 2.2. Identification of Transcriptional Biomarkers of CR in Mouse Liver

**Figure 2:**
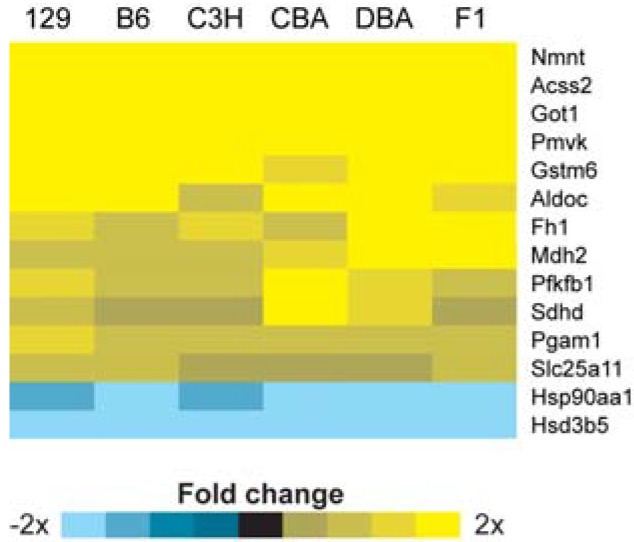
CR-induced change in expression from the microarray data for the 14 biomarkers of CR in mouse liver. The fold change in expression in response to CR is shown for each strain; significant changes are highlighted with yellow or blue fill.

Microarray analysis revealed that of the <20,000 transcripts represented, 76 genes were significantly changed in expression in all six mouse strains (Table A2). We selected 14 genes from this list to be confirmed by reverse transcriptase quantitative PCR (qPCR) in the C57BL6/J strain; selection criteria for qPCR confirmation included a robust fold change in expression (>2-fold change in expression) and a large relative signal intensity from the microarray data. All 14 genes tested by qPCR were confirmed to be significantly modulated by CR (Figure 2).

### 2.3. Identification of Common Pathways Affected by CR in Liver of Multiple Mice Strains

**Figure 3:**
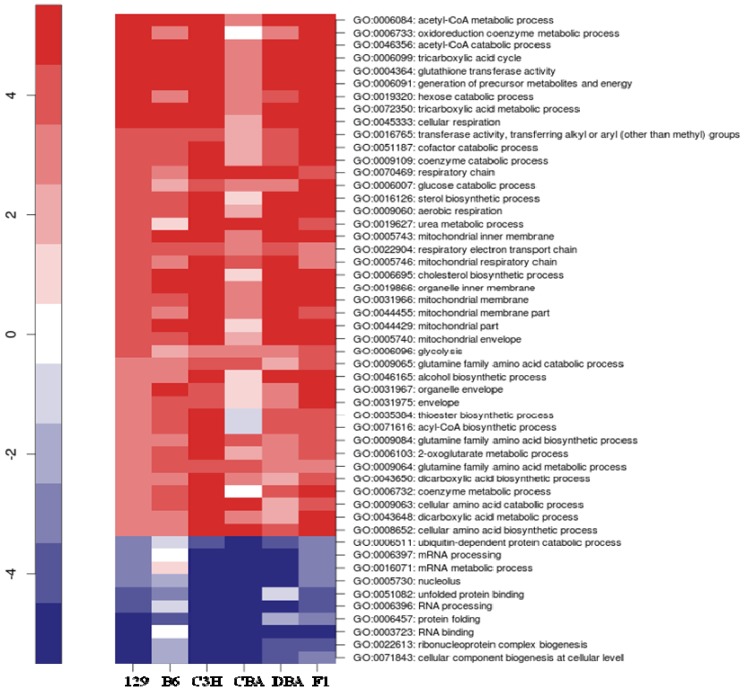
Common pathways for liver CR in multiple mouse strains. Parametric analysis of gene set enrichment (PAGE) identified gene sets significantly modulated by CR (*p* < 0.01, FDR < 0.15) in liver for at least five of the six strains. Each row corresponds to the transcriptional alteration of each gene set with CR. Gene sets up-regulated with CR are shown in red, whereas gene sets down-regulated with CR are shown in blue. Labels indicate the GO ID and GO term of each pathway.

Pathway analysis of the microarray data revealed 24 Gene Ontology (*p* < 0.01 and FDR < 0.15) terms that were significantly modulated in at least five of the six mouse strains (Figure 3). A robust observation was a common induction for multiple strains of genes involved in tricarboxylic cycle (GO:0006099, GO:0072350), mitochondrial (GO:0005743, GO:0005746, GO:00031966, GO:00044429, GO:0044455, GO:0005740), and respiratory chain (GO:0070469, GO:0045271, GO:0022904) metabolism, as well as in specific glucose (GO:0006007), glutamine (GO:0009065, GO:0009084, GO:0009064), glutathione (GO:0004364), cholesterol (GO:0006695) and sterol (GO:0016126), metabolism.

### 2.4. ^1^H NMR Spectroscopy of Blood Plasma on the Six Strains Highlighted a Very Heterogeneous Response in Circulating Lipids Following CR

To characterize the differences in the metabolic profile between Control and CR mice from each of the six strains (10 mice per group), pairwise multivariate models were calculated using OPLS-DA (see Table A3 for model descriptors). Differences (Table 1) in metabolites under CR were mainly driven by changes in high density lipoprotein (HDL), low density lipoprotein (LDL), very low density lipoprotein (VLDL), and poly-unsaturated lipid (PUFA/UFA). Commonly, CR induced lower concentrations of LDL and UFA in 129S1/SvlmJ, C57BL6/J, C3H/HeJ, and JC3F1/J mouse strains. In addition, a lower concentration of HDL was noted in 129S1/SvlmJ and C57BL6/J strains. On the contrary, C3H/HeJ and DBA/2J mice displayed higher concentrations of HDL and, LDL, and both C3H/HeJ and CBA/J strains showed a lower concentration of VLDL under CR. The complexity of the biofluid matrix, which captures the contribution from all the biological compartments, did not enable the identification of robust metabolic signatures of CR across species, but highlighted alterations of lipid handling which is genetic-dependent.

**Table 1:** Summary of metabolites affected by CR (CR *vs*. controls). Differences in peak intensityare listed by order of chemical shift.

Metabolites		Chemical shift	129S1/SvlmJ	C57BL6/J	C3H/HeJ	CBA/J	DBA/2J	JC3F1/J
1	Methyl signal of fatty acids in HDL	0.83 (m)	−0.60	−0.64	0.51	0.09	0.52	−0.37
2	Methyl signal of fatty acids in LDL	0.85 (m)	−0.64	−0.92	0.68	−0.30	0.79	−0.84
3	Methyl signal of fatty acids in VLDL	0.87 (m)	−0.15	−0.49	−0.69	−0.85	0.46	−0.62
5	N-acetyl glycoprotein (NAG) associated resonances	2.04 (s)	−0.83	−0.75	−0.91	−0.67	0.41	−0.55
6	Lipids (FA chain)	2.23 (m)	−0.64	−0.57	−0.68	−0.85	0.37	−0.47
7	Lipids (Polyunsaturated FAs)	2.75 (m)	−0.85	−0.85	−0.63	−0.81	0.51	−0.91
8	Glycerophosphocholine	3.22 (s)	−0.33	−0.73	0.81	0.22	0.81	−0.66
9	Lipids (Unsaturated FAs)	5.30 (m)	−0.88	−0.88	−0.85	0.82	0.71	−0.96

*Key*: Importance of metabolic changes is indicated by their correlation coefficients in the respective pairwise OPLS-DA multivariate analyses between controls and CR group for each mouse strain. The report values correspond to the p (corr) variables. Threshold for significant metabolites was calculated based on two-tailed probability of a Pearson correlation coefficient (*r* = 0.68, *p* < 0.001, *n* = 20), (s = singlets, m = multiplets).

### 2.5. Metabolic Profiling of Urine and Liver Tissues in C57BL6/J Mice

Liver tissue extracts as well as urine samples from C57BL6/J mice were further analyzed using ^1^H-NMR spectroscopy, and CR effect was assessed using chemometric approaches, including pairwise O-PLSA (See Table A4 for model descriptors). Initial analysis of the metabolic signatures (Table 2) ascribed to hydrophilic and lipophilic extracts from liver described a significant alteration of branched chain amino acid, as noted as per accumulation of valine and isoleucine, and a decreased level of the aromatic amino acid phenylalanine. Moreover, increased levels of glutathione were observed in CR. These changes were correlated with an increased level of dimethylglycine, a key metabolic intermediate in the betaine/homcysteine pathway, as well as nicotinurate, and carnosine. CR animals exhibited higher liver concentrations of free and esterified cholesterol, and phospholipids, including phosphatidylcholine and sphyngomyelines, and decreased triacylglycerides. These changes were associated with a remodeling of fatty acid composition, including increased n-3 and n-6 fatty acids C20:3, C20:4, C20:5, and C22:6, retinyl-conjgates, and a decrease in saturated fatty acids. The urine showed changes in several gut microbial metabolites, including an increase in several compounds related to bacterial protein fermentation, (e.g., phenylacetylglycine). This signature of protein digestion was associated with a deep remodelling of host protein/amino acid metabolism, as marked by different excretions of intermediate in the tricarboxylic acid cycle and urea cycle (e.g., α-ketoglutarate and allantoin). A concomitant modulation of the NADP metabolic pathway was also noted with increased concentration of 1-methylnicotinamide, suggesting a differential contribution of TCA and urea cycle for energy production. In addition, some metabolites putatively related to protein synthesis/breakdown were also found in higher urine concentrations of CR animals, including deoxycytidine, taurine, uracil, and pseudouridine.

**Table 2:** Peak integrals (as a.u. = area under) for significantly regulated metabolites in liver tissues, urine from C57BL6/J strain (control *vs*. CR) as detected by ^1^H-NMR.

	Metabolites	Chemical shift	Control Mean ± SD		CR Mean ± SD		OPLS-DA correlation coefficients (*p* corr)	*p*-Value
**Liver hydrophilic fraction**	**Valine**	1.04 (d)	4.54 ± 1.26		7.11 ± 2.12		0.68	<0.001
	**Isoleucine**	1.02 (d)	1.22 ± 2.90		1.84 ± 5.75		0.74	0.001
	**GSH**	2.55(m)	3.55 ± 1.04		6.60 ± 1.94		0.87	<0.001
	**Carnosine**	4.46(m)	2.86 ± 0.68		4.38 ± 1.09		0.73	<0.001
	**Uridine**	5.9 (d)	2.11 ± 0.5		2.03 ± 0.72		−0.79	0.01
	**Phenylalanine**	7.36 (m)	1.26 ± 0.26		1.02 ± 0.34		−0.73	<0.01
	**Nicotinurate**	8.93 (d)	1.34 ± 0.37		2.44 ± 0.52		0.77	<0.001
	**N,N-dimethylglycine**	2.92 (s)	3.91 ± 1.1		6.14 ± 1.37		0.81	<0.001
**Liver Organic Phase**	**Cholesterol (free)**	0.93, 1.01, 1.04 (m)	1.26 ± 0.92		3.14 ± 0.33		0.84	<0.001
	**Cholesterol (total)**	0.69 (s), 0.86–0.88 (dd)	1.54 ± 0.31		3.67 ± 0.31		0.68	<0.001
	**Sphingomyeline**	5.61 (m), 5.35 (m)3.29 (s)	5.98 ± 3.26		18.7 ± 3.36		0.97	<0.001
	**-3 FA**	0.97 (t)	3.50 ± 0.16		5.61 ± 0.13		0.68	<0.001
	**UFA**	5.31 (m),	2.63 ± 0.28		2.05 ± 0.24		−0.69	<0.001
	**Retyl-Conjugate**	6.18 (d)	2.57 ± 1.07		9.37 ± 1.11		0.87	<0.001
	**Fatty acyl chain**	2.34 (m)	4.05 ± 0.23		3.25 ± 0.15		−0.97	<0.001
	**Triacylglycerides (TAG)**	5.09 (m)	5.14 ± 0.56		2.19 ± 0.53		−0.68	<0.001
**Urine**	**L-Fucose**	4.56 (d)	2.90 ± 0.21		3.87 ± 0.42		0.83	<0.001
	**Allantoin**	5.41 (s)	5.10 ± 1.35		9.28 ± 2.30		0.84	<0.001
	**alpha-ketogluterate**	3.01 (t)	1.16 ± 0.11		1.74 ± 0.22		0.88	0.01
	**Taurine**	3.26 (t)	4.63 ± 0.59		7.06± 2.14		0.84	0.001
	**Uracil**	7.54 (d)	5.96 ± 1.15		8.25 ± 1.25		0.72	0.001
	**Pseudouridine**	7.69 (s)	5.01 ± 0.51		6.75 ± 0.96		0.88	0.001
	**N-Methylnicotinamide**	9.28 (s)	2.22 ± 0.49		3.45 ± 0.52		0.76	0.001
	**Phenylacetylglycine**	7.43 (m)	1.47 ± 0.33		2.00 ± 0.24		0.77	0.001
	**Deoxycytidine**	7.83 (d), 6.28 (t), 6.06 (d)	3.95 ± 0.56		6.67 ± 1.72		0.77	<0.001

*Key*: Importance of metabolic changes is indicated by their correlation coefficients in the respective pairwise OPLS-DA multivariate analyses between controls and CR group for each mouse strain. The report values correspond to the *p* (corr) variables. Threshold for significant metabolites was calculated based on two-tailed probability of a Pearson correlation coefficient (*r* = 0.68, *p* < 0.001, *n* = 20), (s = singlet, d = doublets, t = triplets, m = multiplet, dd = dublet of dublet, blue: decrease, orange: increase).

### 2.6. Targeted MS Profiles of Blood Plasma from C57BL6/J Mice

We monitored changes in C57BL6/J mouse blood plasma in the 10 CR and 10 control animals. O-PLS-DA was performed for quantitative information for 163 metabolites, including amino acids, sugars, acyl-carnitines, sphingolipids, and glycerophospholipids, to maximize the separation among CR and control animals. The model descriptors are characterized by high and positive R^2^X, R^2^Y and Q^2^ parameters (Table S3). Based on a variable importance in projection (VIP) threshold (VIP < 1.5) from the seven-fold cross-validated OPLS-DA model together with the V-plot (plot constructed with the VIP value *vs. p* (corr) value of each metabolite), the 10 most influential metabolites responsible for class separation among CR and control are computed [32] (Table 3). Supporting the changes noted in NMR metabolic profiles, CR induced a reduction in circulating levels of polyunsaturated phospholipids, including diacyl and acyl ether phosphocholines. In addition, the concentration of long chain acyl-carnitines (C14:1, C18:1) suggested a modification of long chain fatty acid β-oxidation in mitochondria, whilst decreased concentration of sugars (mainly hexose) further illustrate the depth of the modulation of gluconeogenesis.

**Table 3:** Top 10 most discriminating metabolites in blood serum (mean values ± SD) from the targeted MS on the C57BL6/J strain. Significant differences were assessed by Mann-Whitney U test.

Metabolite (μM/L)	Control Mean ± SD	CR Mean ± SD	*p*-Value	OPLS-DA correlation coefficients (*p* corr)
**PC-O 34:1**	188.5 ± 11.05	62.6 ± 10.5	<0.001	−0.81
**C18:1**	0.44 ± 0.07	0.22 ± 0.06	<0.001	−0.90
**PC 36:1**	24.73 ± 3.65	16.4 ± 1.58	<0.001	−0.86
**PC 34:3**	6.73 ± 1.13	3.49 ± 1.15	0.0001	−0.88
**C14:1**	0.22 ± 0.04	0.14 ± 0.03	0.0002	−0.82
**PC-O 38:0**	3.91 ± 0.92	2.31 ± 0.57	0.0002	−0.89
**PC-O 40:5**	3.48 ± 1.04	2.90 ± 0.53	0.01	−0.80
**PC 40:4**	1.61 ± 0.30	1.24 ± 0.24	0.007	−0.75
**PC 36:5**	3.51 ± 0.92	2.08 ± 0.61	<0.001	−0.83
**Sugars-Hexose**	5996 ± 1.303	3534 ± 1397	0.001	−0.68

*Key*: Importance of metabolic changes ais indicated by their correlation coefficients in the respective pairwise OPLS-DA multivariate analyses between controls and CR group for each mouse strain. The report values correspond to the *p* (corr) variables. Threshold for significant metabolites was calculated based on two-tailed probability of a Pearson correlation coefficient (*r* = 0.68, *p* < 0.001, *n* = 20).

## 3. Discussion

In this study, we report metabolic readouts which are common across mouse strains in response to early CR. Transcriptomic results from the six mouse strains commonly revealed a CR-induced enhancement of mitochondrial metabolic pathways (including fatty acid β-oxidation, modulation of Krebs cycle and overall energy metabolism), a decreased intracellular insulin signaling metabolism, and complementary resistance against oxidative damage. Using complementary metabolic profiling on biofluids and liver, we further describe the metabolic changes in C57BL6/J mice, which support further some of these metabolic adaptive processes to CR.

### 3.1. Complementary Transcriptomics and Metabonomics Reveal CR Effects on Fatty Acid Oxidation, Gluconeogenesis and Cholesterol Metabolism

Our study reveal changes in genes related to acetyl CoA metabolic and catabolic processes (GO: 0006084 and GO: 0046356) in the liver, which illustrates how metabolic response to CR involves mobilization and metabolism of fatty acids. Although fatty acids are metabolized in the mitochondrial matrix, they cannot bypass the mitochondrial membrane without appropriate biochemical processing. Accordingly, the first enzymes involved in processing fatty acids for transport into the mitochondria are a family of acyl CoA synthetases which ligate a molecule of CoA to each fatty acid [33].

Moreover, in the liver of mice subjected to CR, the expression of Acss2 (acyl-CoA synthetase short-chain family member 2) was increased 6.9-fold (*p* < 0.0001). The increased expression of this gene describes an enhanced ability for hepatocytes to process fatty acids for mitochondrial β-oxidation. Interestingly, these physiological changes are mirrored by human studies, as it was hypothesised that inefficient muscle long chain fatty acid (LCFA) β-oxidation is associated with insulin resistance and increased blood plasma fatty acylcarnitines (AC) was reported as a feature specific to type 2 diabetes subjects [34]. AC species are found as a consequence of incompletely oxidized fatty acids with higher rates of substrate use than energy demand, with accumulated acyl-CoA converted to AC that then exits cells and tissues [35]. Our data might suggest reduced entry in and flux through the mitochondrial fatty acid oxidation (FAO) pathway causing a reduced pool of acylcarnitines back into the plasma compartments. Moreover, studies using microarrays have shown that short-term fasting [36] and long-term CR [37] induce a metabolic shift in liver favoring gluconeogenesis over glycolysis, and a very similar pattern was also observed in the current study. In addition, two significant metabolic features associated with hepatic response to CR are representative of differences in mediating the influx of amino acids into the gluconeogenic pathway. The first gene Got1 encodes a cytosolic protein, namely glutamate oxaloacetate transaminase 1 or cytosolic aspartate aminotransferase, which catalyzes the conversion of glutamate into α-ketoglutarate (α-KG). However, the shuttling of α-KG into the mitochondria requires a transport protein located in the mitochondrial inner membrane, namely malate-α-ketoglutarate transporter. The gene coding for this protein (Slc25a11) was as also highlighted as having a significantly up-regulated gene expression in the liver of mice under CR. Therefore, the net effect of these two genes would be an increase in the influx of carbon skeletons into the Krebs cycle which can then be used for gluconeogenesis. The difference in gluconeogenetic metabolic pathways was further illustrated through the observed changes in specific gluconeogenic amino acids and a decreased level of glucose in liver tissues as evidenced by metabonomics. In response to a putative increase in mitochondrial α-KG (above), enzymes within the Krebs cycle and downstream of α-KG entry would be predicted to be up-regulated as well. Our study reveals indeed an up-regulation of set of genes related to TCA metabolic processes (GO: 0072350) and TCA cycle (GO: 0006099). In detail, genes encoding succinate dehydrogenase D (Sdhd) and fumarate hydratase 1 (Fh1) were increased in expression in response to CR (1.6- and 4.2-fold; *p* = 0.0307 and 0.0148, respectively). The net result of increases in the activity of these two enzymes would be an increased production of malate which would then be returned to the cytosol via the malate-α-ketoglutarate transporter Slc25a11 [38] and was identified as a transcriptional biomarker of CR. In response to CR, the pyruvate dehydrogenase complex is inhibited in multiple tissues by activating the family of pyruvate dehydrogenase kinase (Pdk) enzymes [39] the resulting phosphorylation inactivates the pyruvate dehydrogenase complex and inhibits glycolytic glucose metabolism. The gene encoding the mitochondrial isoform of malate dehydrogenase (Mdh2) was increased in expression 1.7-fold in response to CR (*p* = 0.0189), and mitochondrial malate can be subsequently transported to the cytoplasm for gluconeogenesis via Slc25a11. If cytosolic concentrations of malate are indeed increased, as suggested by the regulation of biomarkers of CR, and if malate were converted to phosphoenolpyruvate (PEP) via two known biochemical conversions, this would drive the glycolysis in reverse from PEP through several metabolic conversions to fructose-6-phosphate. Two enzymes in this pathway are phophoglycerate mutase and aldolase, and the genes encoding these proteins were increased in expression in response to CR (Pgam1 = 1.6-fold, *p* = 0.0057; Aldoc = 2.7-fold, *p* = 0.0008). The final transcriptional marker of CR is Pfkfb1 which encodes a single bifunctional protein that responds to circulating hormone levels to shift glycolysis pathways towards glucose catabolism or gluconeogenesis [40]. Circulating glucagon predominates over insulin levels during fasting and CR, and glucagon initiates a signaling cascade that phosphorylates the protein encoded by Pfkfb1 resulting in both a depletion of fructose-2,6-bisphosphate (an allosteric activator of glycolysis) and an accumulation of fructose-6-phosphate which together stimulate gluconeogenic pathways at the hepatic level. Thus, the increased expression of Pfkfb1 (1.7-fold, *p* = 0.0312) combined with relative abundance of glucagon (over insulin) serves to promote hepatic gluconeogenesis during CR. In our study, CR also induced an 8.6-fold increase (*p* < 0.0001) in Pmvk expression, a gene encoding the enzyme phosphomevalonate kinase involved in cholesterol synthesis. Therefore, it is likely that the role of increased Pmvk in response to CR relates to an alternative function of the mevalonate pathway, including synthesis of other sterols and isoprenoids [41]. Transcriptomics data also released that CR has an impact on Hsp90aa1 as well across the six mouse strains. Hsp90aa1 encodes the cytosolic heat shock protein 90 alpha, a molecular chaperone that has specificity for proteins involved in signal transduction. Hsp90aa1 is particularly noted for binding and activating members of the steroid hormone receptor family. In CR mice, Hsp90aa1 expression was changed 1.6-fold (*p* = 0.0067), suggesting a decreased demand for activation of steroid hormone receptors. This possibility is reinforced by the observation of decreased expression of enzymes involved in steroid hormones in response to CR. Moreover, the set of genes related to cholesterol and sterol metabolism were up-regulated (GO: 0006695 and GO: 0016126). Indeed we observed a 5.9-fold change (*p* = 0.0067) in the expression of Hsd3b5 in liver of CR mice, pointing out their role in the response to decreased food intake. It has been suggested that decreased activity of this family of proteins maintains high circulating levels of dehydroepiandrosterone (DHEAS) which has been associated with rhesus monkeys subjected to CR and with longevity in humans. Although the relationship between DHEA and longevity remains controversial, an alternative consequence of decreased Hsd3b5 expression is a decrease in the levels of steroid hormones.

Furthermore, plasma metabotype analysis revealed impact of CR on lipoprotein/lipid metabolism in C57BL6/J mice, as noted with reduced levels of high density lipoproteins (HDL), low density lipoproteins (LDL), and poly-unsaturated fatty acids (PUFA). Here, CR intervention displayed capabilities to modulate the typical lipoprotein atherogenic profile (e.g., high TG/HDL ratio and/or increased LDL concentration), common to metabolic syndrome and/or diabetes, but also in previous studies on CR in lifelong studies in dog [42]. In addition, targeted MS analysis of blood plasma lipids further described a decrease in several polyunsaturated phospholipids, including the following lipid species: PC 34:3, PC 36:1, PC 36:5, PC 40:4. We previously observed similar changes in non-human primates in response to CR, whilst older humans tend to generally show higher PUFAs compared to young, and in obese compared to lean males [43]. Thus, the observation in the current study may be explained in part by the lower fat mass of CR animals. Moreover, increased concentrations of plasma polyunsaturated fatty acids have been implicated in the pathogenesis of chronic diseases [44], with previous reports on higher unsaturated lipids in plasma from obese subjects compared to lean males [43]. Our results showed also an overall decrease on the levels of TAGs in liver from C57BL6/J under CR. The CR-induced decrease in hepatic triglycerides suggests a decreased lipogenesis (reduced FAS expression) and reduced incorporation of triacylglycerols into nascent lipoprotein particles. Despite that no significant changes in circulating levels of VLDL was noted by NMR spectroscopy, the profound remodeling of HDL and LDL levels with CR highlight a different dynamics in lipid exchange from peripherical tissues and the hepatic compartment, which may be involved in greater variations in hepatic lipid metabolism and recycling.

### 3.2. Complementary Transcriptomics and Metabonomics Reveal Impact of CR on Insulin Signaling and Stress Response

There is increasing awareness that the insulin pathway IIS is one of the most potent regulators of aging, by modulating glycolytic processes, lipid metabolism, and the metabolic response to oxidative stress. Indeed, there is a large body of evidence in diverse species showing that longevity is associated with decreased insulin-like signaling [45]. Pathway analysis of the microarray data shows increased liver glucose metabolism with up regulation of set of genes related to glucose catabolic processes (GO:0006007) as well as insulin-like growth factor binding protein 1 (Igfbp1). The latter was significantly increased in five of six strains of mice (not changed in the 129S1/SvImJ, *p* = 0.17) and qPCR analysis indicated that this gene was increased in expression 7.6-fold (*p* < 0.0001) by CR in the C57BL6/J strain (data not shown). An increase in the level of this protein would be predicted to sequester insulin-like growth factor (IGF), thereby decreasing IGF signaling. Interestingly, the expression of several IGF binding proteins was increased in the heart of mice subjected to a similar duration of CR as in this study [46] and in the liver of mice subjected to a 48-hour fast [36]. Thus, IGF binding proteins appear be important transcriptional regulators in response to CR. These changes were further supported by an overall decrease in the concentration of blood and hepatic glucose in C57BL6/J mice under CR, suggesting increased insulin sensitivity and glucose uptake. Another specific signature associated with changes in insulin signaling pathway was also noted via changes in hepatic branched chain amino acids [47] which highlight a change in central energy metabolic and exchanges with peripheral tissues, through BCAA dependent gluconeogenic pathway. Insulin resistance in muscle and fat cells reduces glucose uptake (and also local storage of glucose as glycogen and triglycerides, respectively), whereas insulin resistance in liver cells results in reduced glycogen synthesis and storage and a failure to suppress glucose production and release into the blood.

Following CR intervention, our data also suggest a differential metabolic response to oxidative stress. Here, the set of genes related to glutathione metabolism is up-regulated (GO: 0004364). Specifically, Gstm6 encodes the protein glutathione transferase 6, a member of a family of proteins important in drug metabolism and for defense against oxidative damage. Gstm6 was found to be decreased in expression in liver of diabetic mice [48], whereas Gstm6 was increased in expression 2.6-fold (*p* < 0.0001) by CR. Moreover, increased levels of glutathione and its precursor in the gamma-glutamyl cycle, glutamine, also suggest a different oxidative response induced by CR. In particular, GSH is important in the regulation of the redox state, and a decline in its tissue level has often been considered to be indicative of increased oxidative stress [49]. Further carnosine, found generally in any tissue, is considered to be an anti-aging substance, capable of counteracting oxidative damage and protein glycation [50]. In addition, NMR profiling of liver organic phase revealed increase production of retinly conjugates suggesting an increased protecting role. The liver is the major vitamin A reservoir in mammals, storing up to 80% of the total body vitamin A, and plays therefore a central role in the metabolism, storage and the distribution of retinol to peripheral tissues [51]. The identification of redox homeostasis and its core glutathione as affected pathways in our liver metabonomic analysis, suggesting that reactive oxygen species play a role in the initiation or progression of the CR phenotype. In agreement with these findings, C57BL6/J strain displayed reduction, under CR, in concentration of several acyl-ether (PC-O 34:1, PC-O 38:0, PC-O 40:5), namely plasmalogens. Plasmalogens containing a vinyl ether bond link to the sn-1 aliphatic chain of the glycerol backbone are endogenous antioxidant. Plasmalogens have been implicated in protection of cellular functions against oxidative damage [52] and recent studies displayed that VLDL, LDL and HDL particles are characterized by class-specific ether lipid species composition [53,54].

## 4. Experimental Section

### 4.1. Animals and Dietary Manipulation

All procedures were approved by the Animal Care Committee at the William S. Middleton Memorial Veterans Hospital. Six strains (129S1/SvImJ, C57BL6/J, C3H/HeJ, CBA/J, DBA/2J and /F1) of male mice were purchased at six weeks of age from Jackson Laboratories (Bar Harbor, Maine) and were individually housed in a specific pathogen free facility. Upon arrival, mice were provided with 12.7 kcal (53.1 kJ)·day^−1^ of a pelleted ration of AIN93M diet (Bio-Serv; Frenchtown, New Jersey). At eight weeks of age, half of the mice from each strain were randomly assigned to a control or 25% calorie restricted (CR, 9.44 kcal/39.5kJ·day^−1^) treatment group such that there were 10 mice of each strain in the control or CR group. Details on the feeding regiment and diets used in this study are described elsewhere [55]. Body weight was measured every other week for all mice. For two strains of mice (129S1/SvImJ and C57BL6/J), food intake of the CR group was subsequently decreased to 7.34 kcal (30.7 kJ)·day^−1^ at 16 weeks of age.

### 4.2. Sample Collection

At 20 weeks of age, C57BL6/J mice were individually maintained in Tecniplast metabolic cages for two days to collect urine. At 22 weeks of age, all mice strains were euthanized by cervical dislocation and blood was collected from the body cavity into two heparinized tubes. Tissues from the mice were rapidly dissected, flash-frozen in liquid nitrogen and were stored at −80 °C.

### 4.3. Transcriptomic Analyses

Affymetrix Mouse Genome 430 2.0 microarrays representing 20,341 known genes were used for gene expression profiling; detailed procedures for the microarray analysis are published elsewhere [56]. Briefly, the list of 45,101 probe sets represented on this array was filtered to a list of unique transcripts with an Entrez Gene ID (*i.e*., a gene was only represented once) probe sets representing more than one Entrez Gene ID were removed from the original dataset, and if a gene was represented by more than one probe set, we only retained that probe set having the largest signal intensity when averaged across all Control and CR samples within a strain. For each strain, a gene was considered to be differentially expressed when Two-tailed t-tests *p* < 0.01 using a two tailed t test and when the false discovery rate was < 0.15 [57] were used to identify genes that were differentially expressed in response to CR (*p* < 0.01). Using an Eppendorf realplex2 instrument, the same RNA samples from C57BL6/J mice used for microarray analysis were analyzed by qPCR as described previously [46]. qPCR primers for all genes were purchased from Applied Biosystems. The TATA box binding protein (Tbp) gene was found to be unchanged by CR in all strains according to the microarray data and was therefore used as a housekeeping gene. Of the 19 genes examined by qPCR, only two genes (Foxa3 and Ugt2b35) were not confirmed as being changed by CR. To identify functional classes of genes changed by treatment, we performed parametric analysis of gene set enrichment (PAGE) [58]. PAGE allows for the detection of gene classes that are modulated by an intervention even when there are modest (not statistically significant) but consistent changes in the expression of genes within that functional category (relative to all genes represented on the array. In addition, PAGE generates a z-score indicating if a gene class was activated (z-score < 0) or repressed (z-score < 0) by treatment. We grouped genes into functional classes using the Gene Ontology (GO) hierarchy and only considered those GO terms that were annotated with at least 10 but not more than 1,000 genes per term. Gene functional classes were considered to be significantly altered by treatment at *p* < 0.01. Microarray data have been uploaded to NCBI-GEO [59].

### 4.4. Targeted LC-MS/MS Metabolite Profiling

A targeted LC-MS/MS global metabonomic approach on plasma was used by combining the Biocrates Life Sciences AbsoluteIDQTM kit for plasma samples as previously published [60].

### 4.5. Annotation of Lipid Species

C, acyl-carnitine; LPC, Lysophosphatidylcholines; PC, Phosphatidylcholines; PC-O, 1-O-alkyl-2-acylglycerophosphocholines. Individual lipid species were annotated as follows: [lipid class] [total number of carbon atoms]:[total number of double bonds]. For example, PC 34:4 reflects a phosphatidylcholine species comprising 34 carbon atoms and four double bonds.

### 4.6. Metabolite Profiling-Sample Preparation and 1H NMR Spectroscopic Analysis

Around 5–10 mg of freeze dried and ground tissue was used to extract hydrophilic and lipophilic metabolites applying an adapted Folch procedure [61] as below. Samples were extracted three times with 0.5 mL of a chloroform-methanol solution (2:1, *v:v*). Combined extracts were washed first with 0.5 mL of water and second with 0.5 mL of water-methanol (1:1, *v:v*). The upper hydrophilic phases were collected each time and combined together. Lipophilic and hydrophilic fractions were afterwards evaporated to dryness under nitrogen flow and freeze dried, respectively. Hydrophilic fractions were dissolved in 60 μL of a deuterated phosphate buffer (pH 7.4) containing 1 mM of 3-trimethylsilyl-1-[2,2,3,3,-2H4]-propionate (TSP) as a standard reference (δ = 0.0) and transferred into 1.7 mm NMR tubes. The lipophilic phases were reconstituted in 60 μL of a deuteriated chloroform-methanol solution (2:1, *v:v*) and transferred into 1.7 mm NMR tubes using octamethylcyclotetrasiloxane (OMS) as standard reference (δ = 0.092). All samples were analyzed at ambient temperature (300 K) by ^1^H NMR spectroscopy at 600.13 MHz using a Bruker Avance II NMR spectrometer. For statistical analysis, all NMR spectra were converted into 22 K data points over the range of δ 0.2–10.0 and imported into the MATLAB software (version 7.0; The MathWorks Inc., Natick, MA, USA). The spectra were normalized to a constant total sum of all intensities within the specified range and auto scaled.

### 4.7. Chemometrics

Statistical analysis was performed by Multivariate Data Analysis (MVA) carried out with the Simca-P+ software (version 12.0; Umetrics AB, Umeå, Sweden) and the MATLAB software package (version 7.0; The Mathworks Inc., Natwick, MA). Data import and pre-processing steps for both ^1^H NMR and targeted MS data from C57BL6/J mice groups were done using “in-house” routines written in MATLAB (version 7.11.0, The Mathworks Inc., Natick, MA, USA). Full resolution ^1^H-NMR spectra incorporating data points within the δ 0.4–9.5 region were used for statistical multivariate analysis excluding the water residue signal between δ 4.5–6.5 (urine, plasma, tissue water extract dataset), ethanol δ 1.18 and δ 3.66 (plasma, ethanol residues from antiseptic swabbing contaminated plasma samples during collection), solvent signals (methanol δ = 3.24–3.27 and δ = 4.25–4.60; chloroform δ = 7.35–7.45 for liver tissues organic phase). The supervised Orthogonal PLS discriminant analyses (O-PLS-DA) was applied to the examined biofluids and tissue NMR profiling, and targeted LC-MS/MS metabolite profiling in plasma to maximize the separation between control and CR animals. The validity of the model against overfitting was monitored by computing the cross-validation parameter Q^2^ which represents the predictability of the models and relates to the statistical significance. For the NMR data, differences between samples in the scores’ plot were extracted by using the variable coefficients according to a previously published method [60].Variables correlating with the group separation in the MS data were identified by using the S-plot. It visualizes the variable importance (VIP) score, representing the impact of a single metabolite to the group discrimination of the model [62]. Metabolomics data (C57BL6/J LC-MS/MS μM data concentration of plasma, and C57BL6/J area intensities (a.u.) of urine and tissues ^1^H-NMR data) of representative metabolite signals responsible for class separation is uploaded as a supplemental file (MS Excel).

## 5. Conclusions

The present study provided a comprehensive comparison of the metabolic phenotype at both transcriptomics and metabolomics levels across mice with different genetic backgrounds to identify common metabolic markers affected by CR. Using a system biology approach comprising phenotyping of liver tissue and biofluids, we described the effect of CR across multiple mouse strains (129S1/SvlmJ, C57BL6/J, C3H/HeJ, CBA/J, DBA/2J, JC3F1/J). Overall, our integrated approach commonly described that lipid metabolism between liver and peripheral tissues, oxidative stress response and insulin dependent metabolic pathways are conserved, and are determinant factors involved in CR regulation.

## Appendix

**Table A1:** Body weight for the control and CR strains. Data are reported as mean ± SE values for the body weight gain calculated from Week 8 onwards. Control and CR groups are plotted with dot and square shapes, respectively. Differences of body weight gain were statistically significant between CR and control group were tested by ANOVA and Bonferroni post-tests (^***^ *p* <0.001).

Weeks		Body weight							
		8	10	12	14	16	18	20	22
**129S1/SvlmJ**	Control	22.8 ± 1.26	23.7 ± 1.41	27.4 ± 3.66	29.0 ± 4.17	28.8 ± 4.04	28.1 ± 3.85	28.2 ± 4.35	28.2 ± 4.11
	CR	22.5 ± 0.43	22.8 ± 0.41	25.2 ± 1.33	26.5 ± 1.40	27.0 ± 1.28	25.3 ± 1.32	24.1 ^***^ ± 1.35	23.7 ^***^ ± 1.37
**C57BL6/J**	Control	24.4 ± 2.11	24.1 ± 1.47	26.6 ± 1.98	28.5 ± 2.24	28.4 ± 2.39	28.7 ± 2.39	29.5 ± 2.37	30.5 ± 2.55
	CR	25.6± 1.53	25.6 ± 1.05	26.1 ± 1.01	27.4± 0.96	27.6± 1.13	27.3± 1.01	23.6 ^***^ ± 0.71	23.6 ^***^ ± 0.77
**C3H/HeJ**	Control	25.8± 1.03	28.3 ± 1.14	30.1 ± 1.59	31.6 ± 1.99	32.9 ± 2.83	33.4 ± 3.14	33.6 ± 3.77	33.7 ± 3.73
	CR	24.2 ^***^± 0.82	22.1 ^***^ ± 0.72	22.8 ^***^ ± 0.89	24.2 ^***^ ± 0.76	26.7 ^***^ ± 0.73	28.4 ^***^ ± 0.83	28.4 ^***^ ± 0.73	28.4 ^***^ ± 0.78
**CBA/J**	Control	26.3 ± 0.90	29.3 ± 0.95	29.7 ± 0.99	29.8 ± 1.17	34.5 ± 1.54	35.6 ± 1.41	35.9 ± 1.21	35.9 ± 1.36
	CR	25.1 ^***^ ± 1.27	21.6 ^***^ ± 1.04	21.4 ^***^ ± 0.99	21.4 ^***^ ± 0.96	24.0 ^***^ ± 0.59	25.6 ^***^ ± 1.04	25.7 ^***^ ± 1.45	26.5 ^***^ ± 3.67
**DBA/2J**	Control	24.9 ± 0.79	27.2 ± 1.54	27.8 ± 1.11	30.2 ± 1.85	31.7 ± 2.09	31.9 ± 2.27	32.1 ± 2.24	31.4 ± 2.48
	CR	24.4 ± 1.05	22.1 ^***^ ± 1.11	22.8 ^***^ ± 1.22	24.1 ^***^ ± 1.36	25.5 ^***^ ± 1.41	25.9 ^***^ ± 1.53	25.8 ^***^ ± 1.55	25.0 ^***^ ± 1.54
**JC3F1/J**	Control	27.8 ± 0.79	33.6 ± 0.55	35.7 ± 0.60	36.6 ± 1.03	36.1 ± 0.99	36.3 ± 0.94	38.3 ± 1.22	37.5 ± 1.04
	CR	28.4 ± 0.94	26.2 ^***^ ± 0.67	27.3 ^***^ ± 1.05	28.0 ^***^ ± 1.11	27.5 ^***^ ± 1.24	27.2 ^***^ ± 1.45	29.2 ^***^ ± 1.59	28.4 ^***^ ± 1.47

**Table A2:** Number of genes changed in expression for each strain. Values reported are p value and fold change (FC).

Gene Symbol	Gene Title	*p*-value (129)	FDR (129)	FC (129)	*p*-value (B6)	FDR (B6)	FC (B6)	*p*-value (C3H)	FDR (C3H)	FC (C3H)	*p*-value (CBA)	FDR (CBA)	FC (CBA)	*p*-value (DBA)	FDR (DBA)	FC (DBA)	*p*-value (F1)	FDR (F1)	FC (F1)
4931406C07Rik	RIKEN cDNA 4931406C07 gene	0.000	0.003	−1.380	0.001	0.030	−1.290	0.004	0.071	−1.290	0.000	0.000	−1.900	0.000	0.003	−1.680	0.000	0.009	−1.400
9530008L14Rik	RIKEN cDNA 9530008L14 gene	0.000	0.002	−2.010	0.000	0.013	−1.430	0.001	0.031	−1.310	0.000	0.000	−1.840	0.000	0.008	−1.890	0.000	0.001	−1.390
Acadm	Acyl-Coenzyme A dehydrogenase, medium chain	0.000	0.000	−1.850	0.000	0.003	−1.430	0.001	0.030	−1.270	0.009	0.033	−1.210	0.002	0.088	−1.510	0.000	0.007	−1.430
Acss2	acyl-CoA synthetase short-chain family member 2	0.006	0.099	4.230	0.000	0.014	4.340	0.000	0.002	3.600	0.000	0.002	2.400	0.000	0.001	5.640	0.000	0.001	6.270
Adh4	alcohol dehydrogenase 4 (class II), pi polypeptide	0.000	0.011	−1.570	0.002	0.035	−1.460	0.007	0.106	−1.310	0.000	0.002	−2.250	0.000	0.034	−1.800	0.000	0.001	−1.640
Aldoc	aldolase C, fructose-bisphosphate	0.000	0.000	4.140	0.000	0.001	2.230	0.004	0.074	1.590	0.000	0.000	2.190	0.000	0.001	2.080	0.002	0.062	1.780
Asl	argininosuccinate lyase	0.000	0.003	1.700	0.003	0.048	1.450	0.000	0.001	1.670	0.000	0.000	2.740	0.000	0.004	1.920	0.002	0.059	1.660
Ass1	argininosuccinate synthetase 1	0.000	0.005	2.330	0.001	0.026	1.670	0.000	0.003	2.010	0.000	0.000	5.340	0.000	0.000	2.950	0.001	0.032	2.010
Atp5g1	ATP synthase, H+ transporting, mitochondrial F0 complex, subunit c1 (subunit 9)	0.001	0.022	1.600	0.003	0.043	1.510	0.000	0.000	1.410	0.000	0.000	1.770	0.000	0.017	1.830	0.000	0.006	1.520
Chrna2	cholinergic receptor, nicotinic, alpha polypeptide 2 (neuronal)	0.000	0.001	−5.230	0.000	0.002	−3.170	0.000	0.003	−2.670	0.000	0.000	−4.260	0.002	0.078	−4.090	0.002	0.052	−1.960
D16H22S680E	DNA segment, Chr 16, human D22S680E, expressed	0.000	0.016	1.640	0.000	0.000	2.140	0.000	0.012	1.570	0.000	0.000	1.760	0.000	0.029	1.700	0.001	0.029	1.310
Dhcr7	7-dehydrocholesterol reductase	0.000	0.018	2.970	0.001	0.023	2.430	0.000	0.013	2.950	0.003	0.014	1.920	0.000	0.029	2.870	0.000	0.005	3.160
Dlat	dihydrolipoamide S-acetyltransferase (E2 component of pyruvate dehydrogenase complex)	0.001	0.026	1.530	0.000	0.009	1.470	0.001	0.022	1.830	0.000	0.004	−1.710	0.000	0.029	1.960	0.001	0.039	1.970
Echdc1	enoyl Coenzyme A hydratase domain containing 1	0.005	0.090	1.780	0.000	0.002	1.790	0.002	0.051	1.680	0.000	0.000	−2.410	0.000	0.024	1.890	0.005	0.100	1.970
Eci1	enoyl-Coenzyme A delta isomerase 1	0.000	0.000	−2.720	0.000	0.000	−1.800	0.002	0.049	−1.440	0.005	0.022	1.310	0.000	0.030	−1.620	0.004	0.087	−1.520
Fbxo18	F-box protein 18	0.000	0.009	1.590	0.000	0.013	1.410	0.000	0.010	1.400	0.000	0.000	2.160	0.004	0.121	1.260	0.000	0.011	1.430
Fdps	farnesyl diphosphate synthetase	0.001	0.038	6.470	0.001	0.023	5.310	0.000	0.011	3.510	0.009	0.033	1.920	0.000	0.000	7.660	0.000	0.009	4.870
Fh1	fumarate hydratase 1	0.000	0.021	1.740	0.000	0.011	1.440	0.000	0.001	1.910	0.002	0.011	1.430	0.000	0.004	2.080	0.000	0.003	2.010
Foxa3	forkhead box A3	0.002	0.042	−1.940	0.000	0.001	−1.980	0.000	0.005	−2.580	0.000	0.002	−1.820	0.000	0.011	−2.020	0.000	0.001	−2.440
Gls2	glutaminase 2 (liver, mitochondrial)	0.000	0.001	3.080	0.000	0.000	2.430	0.000	0.000	2.210	0.000	0.000	2.700	0.000	0.025	2.050	0.000	0.003	2.080
Got1	glutamate oxaloacetate transaminase 1, soluble	0.000	0.009	3.130	0.000	0.001	2.310	0.000	0.000	4.230	0.000	0.002	4.020	0.004	0.121	4.300	0.000	0.000	4.130
Gsta4	glutathione S-transferase, alpha 4	0.000	0.000	2.510	0.000	0.000	2.690	0.003	0.070	1.400	0.000	0.000	1.910	0.000	0.035	1.510	0.000	0.018	1.760
Gstm2	glutathione S-transferase, mu 2	0.001	0.040	2.110	0.000	0.007	1.680	0.002	0.054	1.800	0.001	0.006	1.730	0.000	0.004	3.020	0.000	0.001	2.680
Gstm6	glutathione S-transferase, mu 6	0.001	0.023	2.030	0.000	0.002	2.030	0.000	0.015	2.300	0.002	0.009	1.840	0.000	0.001	3.030	0.000	0.000	2.880
Heatr1	HEAT repeat containing 1	0.000	0.001	2.110	0.000	0.011	1.520	0.000	0.000	1.690	0.002	0.009	1.370	0.000	0.003	1.550	0.000	0.001	1.750
Hgd	homogentisate 1, 2-dioxygenase	0.006	0.101	1.340	0.000	0.011	1.280	0.000	0.000	1.630	0.000	0.000	2.140	0.000	0.022	1.820	0.000	0.002	1.760
Hsd3b5	hydroxy-delta-5-steroid dehydrogenase, 3 beta- and steroid delta-isomerase 5	0.009	0.129	−2.140	0.003	0.043	−13.12	0.000	0.002	−3.790	0.000	0.002	−2.400	0.000	0.003	−5.730	0.000	0.019	−12.07
Hsp90aa1	heat shock protein 90, alpha (cytosolic), class A member 1	0.002	0.044	−1.790	0.000	0.001	−2.030	0.000	0.016	−1.740	0.000	0.000	−3.640	0.006	0.149	−2.390	0.000	0.008	−2.220
Ikbkg	inhibitor of kappaB kinase gamma	0.000	0.002	−1.480	0.000	0.000	−1.640	0.005	0.085	−1.370	0.000	0.000	−1.750	0.002	0.080	−1.690	0.002	0.050	−1.380
Klc4	kinesin light chain 4	0.000	0.001	1.750	0.000	0.001	1.460	0.000	0.003	1.490	0.001	0.004	1.410	0.000	0.032	1.550	0.000	0.006	1.370
Lonp2	lon peptidase 2, peroxisomal	0.000	0.000	−2.040	0.000	0.000	−1.950	0.001	0.028	−1.450	0.001	0.005	−1.380	0.000	0.004	−1.790	0.000	0.002	−1.550
Mdh2	malate dehydrogenase 2, NAD (mitochondrial)	0.003	0.063	1.540	0.002	0.032	1.540	0.000	0.017	1.590	0.000	0.000	1.810	0.004	0.126	2.030	0.001	0.043	2.080
Mgll	monoglyceride lipase	0.000	0.000	−2.260	0.000	0.000	−2.730	0.000	0.007	−2.350	0.000	0.000	−2.870	0.000	0.010	−2.000	0.000	0.001	−2.210
Mmd	monocyte to macrophage differentiation-associated	0.000	0.000	−1.760	0.001	0.023	−1.270	0.004	0.075	−1.320	0.000	0.002	−1.780	0.001	0.064	−1.300	0.000	0.001	−1.570
Mthfd1	methylenetetrahydrofolate dehydrogenase (NADP+ dependent), methenyltetrahydrofolate cyclohydrolase, formyltetrahydrofolate synthase	0.003	0.068	1.510	0.000	0.008	1.680	0.000	0.022	1.410	0.000	0.000	2.820	0.001	0.054	1.740	0.009	0.137	1.380
Nadsyn1	NAD synthetase 1	0.000	0.008	1.790	0.000	0.008	1.610	0.004	0.072	1.380	0.000	0.000	1.570	0.003	0.099	1.610	0.000	0.024	1.360
Ndufs8	NADH dehydrogenase (ubiquinone) Fe-S protein 8	0.000	0.000	1.530	0.008	0.085	1.270	0.001	0.025	1.260	0.006	0.025	−1.210	0.004	0.126	1.310	0.000	0.016	1.260
Ngef	neuronal guanine nucleotide exchange factor	0.000	0.002	2.140	0.000	0.000	2.100	0.000	0.003	1.470	0.000	0.000	1.930	0.003	0.094	1.360	0.001	0.027	1.340
Nkiras2	NFKB inhibitor interacting Ras-like protein 2	0.009	0.129	1.460	0.001	0.023	1.340	0.000	0.009	1.330	0.000	0.000	1.890	0.001	0.051	1.430	0.000	0.010	1.490
Nnmt	nicotinamide N-methyltransferase	0.000	0.001	12.500	0.000	0.003	2.890	0.000	0.020	20.330	0.000	0.001	4.000	0.000	0.002	18.800	0.000	0.011	5.630
Nol8	nucleolar protein 8	0.002	0.043	−1.550	0.003	0.044	−1.390	0.000	0.010	−1.650	0.000	0.002	−1.680	0.003	0.094	−1.660	0.001	0.027	−1.730
Pa2g4	proliferation-associated 2G4	0.000	0.003	−1.400	0.000	0.001	−2.010	0.000	0.011	−1.620	0.000	0.000	−1.610	0.001	0.065	−1.450	0.000	0.005	−1.520
Paox	polyamine oxidase (exo-N4-amino)	0.000	0.014	2.960	0.000	0.008	2.930	0.000	0.002	2.240	0.000	0.000	1.530	0.000	0.001	2.390	0.000	0.000	2.480
Paqr9	progestin and adipoQ receptor family member IX	0.000	0.003	−4.730	0.000	0.000	−3.590	0.001	0.025	−1.610	0.000	0.000	−3.550	0.005	0.131	−3.970	0.000	0.008	−1.770
Pcca	propionyl-Coenzyme A carboxylase, alpha polypeptide	0.000	0.018	1.510	0.000	0.005	1.330	0.000	0.001	1.390	0.000	0.000	1.840	0.001	0.045	1.450	0.000	0.009	1.480
Pctp	phosphatidylcholine transfer protein	0.000	0.000	−3.120	0.000	0.000	−1.610	0.001	0.040	−1.850	0.000	0.001	−2.950	0.000	0.027	−1.960	0.001	0.024	−1.380
Pdilt	protein disulfide isomerase-like, testis expressed	0.000	0.003	−3.470	0.001	0.023	−2.850	0.000	0.018	−2.100	0.000	0.000	−3.690	0.003	0.102	−2.180	0.009	0.135	−1.580
Pdzk1	PDZ domain containing 1	0.000	0.002	−1.550	0.001	0.028	−1.410	0.000	0.002	−1.470	0.004	0.018	−1.300	0.001	0.061	−1.480	0.000	0.009	−1.310
Pemt	phosphatidylethanolamine N-methyltransferase	0.002	0.041	1.430	0.003	0.043	1.430	0.000	0.000	1.620	0.002	0.012	1.460	0.001	0.054	1.640	0.000	0.005	1.760
Pfkfb1	6-phosphofructo-2-kinase/fructose-2,6-biphosphatase 1	0.000	0.000	1.780	0.009	0.093	1.370	0.001	0.041	1.530	0.000	0.000	2.560	0.000	0.024	1.820	0.002	0.049	1.470
Pgam1	phosphoglycerate mutase 1	0.000	0.014	1.680	0.000	0.003	1.420	0.000	0.005	1.390	0.003	0.016	1.400	0.000	0.005	1.640	0.000	0.003	1.500
Pgk1	phosphoglycerate kinase 1	0.001	0.034	1.450	0.000	0.002	1.180	0.000	0.006	1.230	0.000	0.000	1.700	0.000	0.036	1.410	0.001	0.044	1.290
Pla2g16	phospholipase A2, group XVI	0.000	0.006	2.180	0.002	0.033	1.840	0.000	0.003	1.590	0.000	0.000	2.210	0.000	0.038	2.060	0.000	0.005	1.930
Pmvk	phosphomevalonate kinase	0.002	0.044	2.380	0.001	0.019	4.340	0.000	0.010	3.110	0.001	0.005	1.960	0.000	0.001	4.520	0.000	0.003	4.000
Ppif	peptidylprolyl isomerase F (cyclophilin F)	0.000	0.004	1.790	0.001	0.024	1.390	0.001	0.027	1.320	0.000	0.000	2.080	0.001	0.048	1.580	0.000	0.006	1.330
Ppp1r3g	protein phosphatase 1, regulatory (inhibitor) subunit 3G	0.000	0.013	2.540	0.000	0.001	8.450	0.000	0.018	3.460	0.000	0.000	3.080	0.001	0.066	2.750	0.001	0.045	4.010
Retsat	retinol saturase (all trans retinol 13,14 reductase)	0.000	0.000	−3.880	0.000	0.000	−1.590	0.001	0.026	−1.640	0.007	0.027	−1.400	0.001	0.047	−1.820	0.004	0.091	−1.450
Rilp	Rab interacting lysosomal protein	0.009	0.128	1.410	0.000	0.010	1.350	0.007	0.105	1.420	0.000	0.000	2.060	0.000	0.008	1.630	0.002	0.049	1.310
Ropn1l	ropporin 1-like	0.000	0.004	2.030	0.000	0.012	1.590	0.007	0.108	1.520	0.003	0.013	1.650	0.000	0.014	1.950	0.006	0.109	1.420
Sdhd	succinate dehydrogenase complex, subunit D, integral membrane protein	0.001	0.039	1.570	0.003	0.049	1.250	0.000	0.002	1.270	0.000	0.000	1.990	0.000	0.004	1.710	0.001	0.027	1.340
Sds	serine dehydratase	0.000	0.008	2.080	0.000	0.000	2.620	0.000	0.009	2.650	0.001	0.009	2.210	0.002	0.089	2.190	0.000	0.002	2.850
Selenbp1	selenium binding protein 1	0.000	0.001	2.430	0.000	0.000	2.240	0.000	0.005	1.800	0.003	0.015	1.810	0.004	0.121	1.530	0.000	0.000	1.800
Slc25a11	solute carrier family 25 (mitochondrial carrier oxoglutarate carrier), member 11	0.000	0.003	1.530	0.000	0.005	1.390	0.004	0.081	1.240	0.010	0.035	1.180	0.000	0.029	1.290	0.000	0.002	1.460
Slc25a12	solute carrier family 25 (mitochondrial carrier, Aralar), member 12	0.001	0.022	1.810	0.000	0.001	1.800	0.003	0.064	1.420	0.000	0.000	1.920	0.000	0.006	1.780	0.001	0.029	1.510
Slc25a20	solute carrier family 25 (mitochondrial carnitine/acylcarnitine translocase), member 20	0.000	0.001	−2.090	0.000	0.003	−1.590	0.002	0.052	−1.500	0.000	0.001	−2.070	0.001	0.053	−1.310	0.000	0.003	−1.620
Slc2a4	solute carrier family 2 (facilitated glucose transporter), member 4	0.006	0.102	2.460	0.000	0.002	3.060	0.007	0.111	1.750	0.004	0.018	2.000	0.000	0.018	2.410	0.000	0.003	2.820
Slc3a1	solute carrier family 3, member 1	0.000	0.002	3.170	0.000	0.001	1.740	0.001	0.032	2.740	0.000	0.000	3.020	0.000	0.009	2.100	0.000	0.001	2.630
Stt3b	STT3, subunit of the oligosaccharyltransferase complex, homolog B (S. cerevisiae)	0.000	0.002	−1.310	0.000	0.000	−1.410	0.001	0.027	−1.220	0.000	0.000	−1.440	0.003	0.106	−1.330	0.000	0.006	−1.260
Tsc22d1	TSC22 domain family, member 1	0.000	0.004	−3.210	0.000	0.001	−2.840	0.007	0.111	−1.660	0.000	0.002	−2.940	0.000	0.006	−2.640	0.004	0.088	−1.610
Tubb4b	tubulin, beta 4B class IVB	0.000	0.000	−2.190	0.000	0.000	−2.220	0.000	0.022	−1.680	0.001	0.007	−1.410	0.005	0.141	−1.510	0.004	0.087	−1.500
Txndc15	thioredoxin domain containing 15	0.001	0.024	−1.350	0.001	0.018	−1.280	0.000	0.017	−1.170	0.000	0.000	−1.750	0.003	0.096	−1.380	0.000	0.006	−1.490
Ube2d2a	ubiquitin-conjugating enzyme E2D 2A	0.002	0.048	−1.260	0.000	0.001	−1.310	0.001	0.033	−1.250	0.000	0.000	−1.930	0.003	0.094	−1.210	0.002	0.047	−1.350
Ube2e2	ubiquitin-conjugating enzyme E2E 2	0.000	0.000	−2.060	0.000	0.002	−1.780	0.003	0.066	−1.370	0.000	0.000	−2.310	0.000	0.009	−1.860	0.000	0.023	−1.420
Ubxn4	UBX domain protein 4	0.000	0.000	−1.660	0.000	0.000	−2.200	0.000	0.001	−1.500	0.010	0.035	−1.260	0.000	0.004	−1.720	0.000	0.003	−1.410
Ugt2b35	UDP glucuronosyltransferase 2 family, polypeptide B35	0.000	0.008	−1.660	0.000	0.003	−1.520	0.000	0.004	−1.840	0.000	0.000	−2.630	0.001	0.062	−2.000	0.000	0.007	−1.690
Vnn1	vanin 1	0.000	0.000	−4.960	0.000	0.004	−2.160	0.001	0.026	−4.670	0.000	0.000	−5.050	0.000	0.014	−3.680	0.000	0.000	−3.100

**Table A3:** O-PLS-DA model summary for discriminating plasma metabolic profiles based on targeted MS profiling and NMR analysis on the six tested animal strains.

Models	Model descriptor	129S1/SvlmJ	C57BL6/J	C3H/HeJ	CBA/J	DBA/2J	JC3F1/J
NMR Diffusion edited	Q^2^Y	0.22	0.60	0.33	0.50	0.19	0.64
plasma	R^2^X	0.17	0.22	0.11	0.12	0.13	0.22
spectra	R^2^Y	0.61	0.77	0.79	0.86	0.76	0.79
Targeted MS Profiling	Q^2^Y R^2^X R^2^Y	-	0.59 0.24 0.74	-	-	-	-

**Table A4:** O-PLS-DA model summary for discriminating for ^1^H-NMR profiling of urine, liver tissues for the C57BL6/J strain.

Models	Model descriptor	C57BL6/J
NMR urine	Q^2^Y R^2^X R^2^Y	0.910.91
NMR water phase liver tissue	Q^2^Y R^2^X R^2^Y	0.42
NMR organic phase liver tissue	Q^2^Y R^2^X R^2^Y	0.92

## Supplementary Files

Supplementary File 1 Supplementary (XLSX, 26 KB)
